# AIOLOS Variants Causing Immunodeficiency in Human and Mice

**DOI:** 10.3389/fimmu.2022.866582

**Published:** 2022-04-04

**Authors:** Motoi Yamashita, Tomohiro Morio

**Affiliations:** Department of Pediatrics and Developmental Biology, Graduate School of Medical and Dental Sciences, Tokyo Medical and Dental University, Tokyo, Japan

**Keywords:** AIOLOS, IKZF3, IKAROS, IKZF transcription factor, inborn errors of immunity

## Abstract

AIOLOS is encoded by *IKZF3* and is a member of the IKAROS zinc finger transcription factor family. Heterozygous missense variants in the second zinc finger of AIOLOS have recently been reported to be found in the families of patients with inborn errors of immunity. The AIOLOS^G159R^ variant was identified in patients with B-lymphopenia and familial Epstein–Barr virus-associated lymphoma. Early B-cell progenitors were significantly reduced in the bone marrow of patients with AIOLOS^G159R^. Another variant, AIOLOS^N160S^ was identified in the patients presented with hypogammaglobulinemia, susceptibility to *Pneumocystis jirovecii* pneumonia, and chronic lymphocytic leukemia. Patients with AIOLOS^N160S^ had mostly normal B cell counts but showed increased levels of CD21^lo^ B cells, decreased CD23 expression, and abrogated CD40 response. Both variants were determined to be loss-of-function. Mouse models harboring the corresponding patient’s variants recapitulated the phenotypes of the patients. AIOLOS is therefore a novel disease-causing gene in human adaptive immune deficiency.

## Introduction

AIOLOS, encoded by the *IKZF3* gene, is a member of the IKAROS zinc finger (IKZF) transcription factor family. AIOLOS was first discovered as a binding partner of the pioneer molecule of the IKZF family IKAROS (1). The early studies of Aiolos-deficient mice revealed that Aiolos regulates B cell activation and maturation, Th17 differentiation, and natural killer (NK) cell maturation (2–4). Ikaros and Aiolos cooperatively regulate the expression of λ5, a pre-B cell receptor component, in pre-B cells (5). They also suppress c-Myc expression in pre-B cells, inhibiting pre-B cell proliferation (6). Aiolos, together with Ikaros, is also involved in T cell development. They regulate CD8α expression in thymocytes and regulate Bcl-6 expression in follicular helper T cells (7, 8).

**Figure 1:**
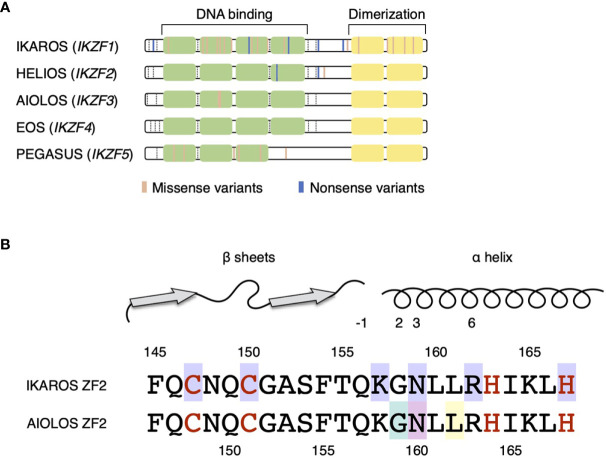
IKZF protein structures. **(A)** DNA-binding zinc fingers (ZF1–4) and dimerizing zinc fingers (ZF5–6) are represented by light green and yellow boxes, respectively. The colored lines represent the germline variants reported in IEIs (IKAROS, HELIOS, and AIOLOS) and hereditary thrombocytopenia (PEGASUS). The exon junctions are indicated with dashed lines. **(B)** Amino acid sequences of the ZF2 of IKAROS and AIOLOS are presented. The numbers indicate the positions of amino acids, whereas red letters represent the amino acids interacting with zinc ions. Above, the secondary structures of ZF2 are shown with the numbers represent amino acid positions in reference to the beginning of α helix. Missense variants of amino acids with purple background are known to be the causes of IKAROS deficiency. Missense variants of G159 (light green background) and N160 (pink background) are known to cause immunodeficiency. The arginine substitution of L162 (yellow background) is known as the hotspot somatic mutation in CLL.

IKZF family transcription factors share the N-terminal C2H2 zinc finger (ZF) domain that is required for DNA binding and the C-terminal C2H2 ZF domain that mediates homo- and heterodimerization (**Figure 1A**) (9). IKZF proteins comprise the nucleosome remodeling, histone deacetylase complex and polycomb repressive complex 2 in the form of homo- and heterodimers (10, 11). They modulate the chromatin status of the target genes and mediate the activating as well as the repressive effects. Moreover, these proteins play pivotal roles in maintaining and developing various hematopoietic cells, but are especially important for the development of lymphocytes.

IKAROS was the first member of IKZF family protein to be discovered as the causative gene in the patients with inborn errors of immunity (IEI) (12–14). Heterozygous loss-of-function variants of *IKZF1* result in IKAROS deficiency, which is characterized by a reduction in the levels of B cells and hypogammaglobulinemia often associated with recurrent sinopulmonary infections and other infections (9). Autoimmunity and hematologic malignancies are frequently observed to occur. In particular, IKAROS^N159S/T^ variants exert dominant-negative effect against wild-type IKAROS (14–16). Even though the wide range of clinical phenotypes is observed in the patients with IKAROS loss-of-function variants, the patients with IKAROS N159 missense variants present with a profound combined immunodeficiency phenotype (9, 17). All patients developed *Pneumocystis jirovecii* pneumonia (PjP) in the first years of life. Various bacterial, viral, fungal, and parasitic infections were also observed. The patients with IKAROS N159 missense variants manifest profound B-lymphopenia associated with severe hypogammaglobulinemia. T cells were present but manifested predominantly naive phenotype, and Th polarization defect was observed. The heterozygous and homozygous germline variants of HELIOS have been recently identified in the patients with recurrent sinopulmonary infections, chronic *Candida* infection, hypogammaglobulinemia, and lymphoma (18, 19). Heterozygous HELIOS variants have also recently been reported in patients with autoimmune diseases such as systemic lupus erythematosus, and Epstein–Barr virus (EBV)-associated hemophagocytic lymphohistiocytosis (20). In addition, heterozygous PEGASUS variants have been identified in a cohort with hereditary thrombocytopenia (21).

Somatic mutations of AIOLOS have been reportedly found in hematologic malignancies, including B-cell acute lymphoblastic leukemia (ALL) and chronic lymphocytic leukemia (CLL) (22–25). Two recent studies revealed the germline variants of AIOLOS in human diseases (**Figure 1B**) (26, 27). Therefore, we reviewed the clinical characteristics and murine models of the AIOLOS variants in these studies.

## AIOLOS Variants Associated With Human IEI

### AIOLOS G159R Variant

**Figure 2:**
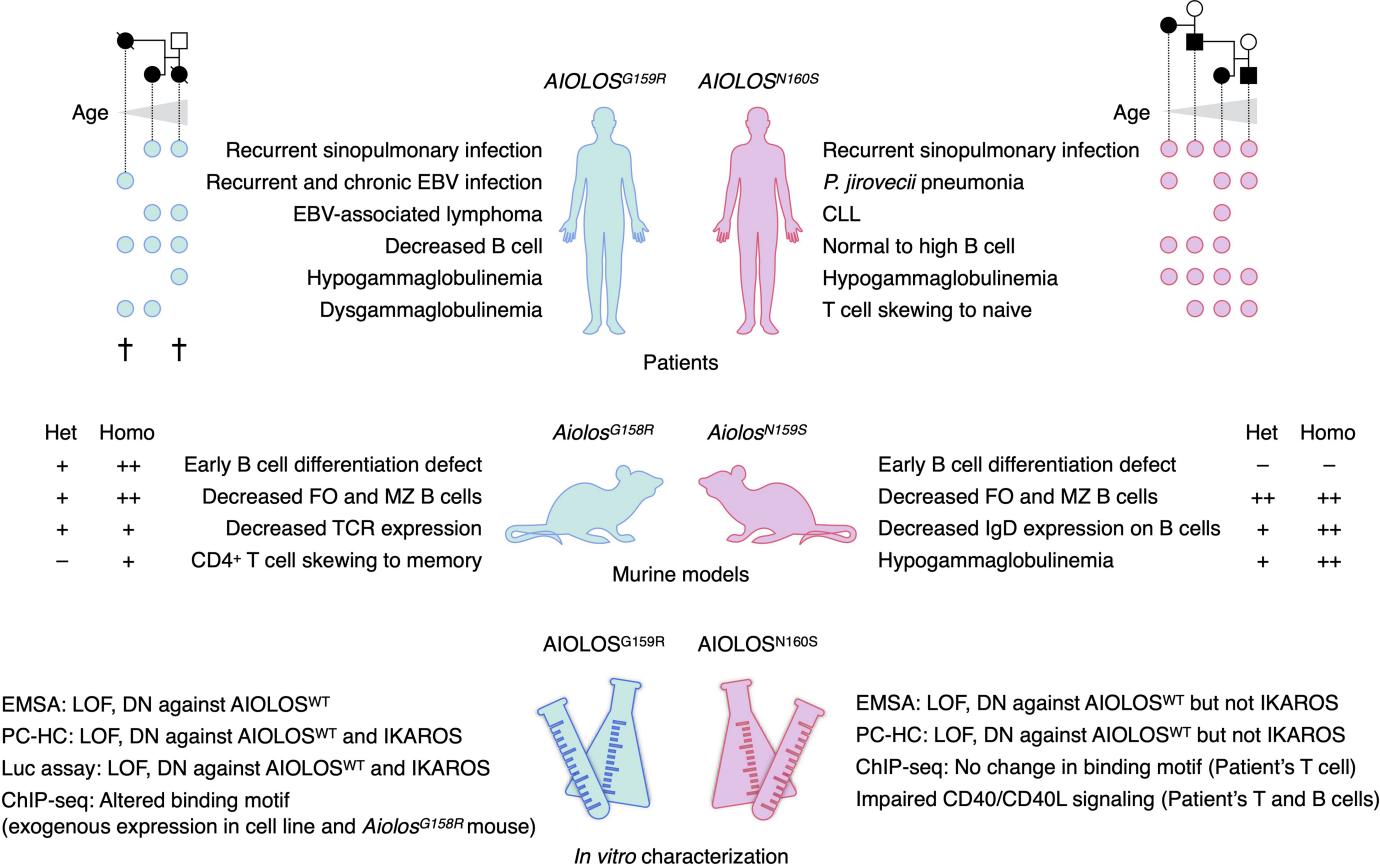
Summary of patients, murine models, and *in vitro* studies of AIOLOS variants. Circles indicate the patients who presented with the indicated phenotypes. Each column of the circle represents each patient, sorted by the age of the patients. Pedigrees of the study patients are presented above. Two patients with *AIOLOS^G159R^* had died at the time of the study. The phenotypes of both heterozygous (Het) and homozygous (Homo) mice were presented. EBV, Epstein–Barr virus; CLL, chronic lymphocytic leukemia; FO, follicular; MZ, marginal zone; TCR, T-cell receptor; EMSA, electrophoretic mobility shift assay; DN, dominant-negative; PC-HC, pericentromeric heterochromatin foci formation assay; LOF, loss-of-function.

Yamashita et al. reported the case of three patients in a family with profound B-lymphopenia and familial lymphoma (presented in **Figure 2** and **Table 1**) (26). The index patient was a 42-year-old woman with recurrent sinopulmonary infections since childhood, requiring home oxygen therapy. She presented with splenomegaly in her 30s and subsequently developed cervical non-Hodgkin lymphoma (follicular lymphoma and EBV-positive diffuse large B-cell lymphoma [DLBCL]) at the age of 42 years. Laboratory studies revealed hypogammaglobulinemia, pancytopenia, and profound reduction in B-cell levels. After chemotherapy for lymphoma, she underwent bone marrow transplantation (BMT) and subsequently died of multiple organ failure after transplantation. Her son presented with the same clinical characteristics: recurrent sinopulmonary infection and adult-onset B-cell lymphoma (EBV-positive DLBCL). Additionally, he experienced recurrent perianal abscesses in his childhood. Moreover, his B-cell levels in the peripheral blood lymphocytes were remarkably low. He underwent allogeneic BMT and survived. Conversely, the daughter of the index patient exhibited susceptibility to EBV infection. She experienced recurrent infectious mononucleosis due to EBV at 4 years of age, repeated EBV-associated hemophagocytic syndrome, and contracted chronic active EBV disease (CAEBV) in the following year. B-lymphopenia was noted upon examination. She died of gastrointestinal bleeding associated with CAEBV at the age of 6 years. Autoimmunity was not observed in any of the patients with the AIOLOS^G159R^ variant.

**Table 1:** Summary of the patients and murine models of AIOLOS variants.

	AIOLOS^G159R^/Aiolos^G158R^		AIOLOS^N160S^/Aiolos^N159S^	
	Patients	Mouse	Patients	Mouse
**Clinical phenotypes**				
**Infection**	Recurrent sinopulmonary infection (2/3)		Recurrent sinopulmonary infection (3/4)	
	Recurrent EBV-IM (1/3)		*P. jirovecii* pneumonia (3/4)	
	Recurrent EBV-VAHS (1/3)		Bacterial meningitis (1/4), and sepsis (1/4)	
	CAEBV (1/3)		Extensive cutaneous warts (1/4)	
			*M. avium* complex lung disease (1/4)	
**Autoimmunity**	None		None	
**Malignancy**	EBV-associated lymphoma (2/3)		CLL (1/4)	
			Metastatic melanoma (1/4)	
**Immunological phenotypes**				
**B cells**	↓ B cell (3/3)	Differential blockade in early B-cell progenitors	→ to ↑ B cell (3/4), and ↓ B cell (1/4)	Modest ↑ of B cells in heteroygous, and ↓ in homozygous mice of PB
	↓ B cell-lineage commited progenitor in BM (1/1 evaluated)	↓ splenic B cells	↑ CD21lo B cells, with minimal CD23 expressions (4/4)	↓ FO B cells
	Hypogammaglobulinemia (1/3)	↓ FO B cells and MZ B cells	↓ memory B cells and plasmablasts differentiation (4/4)	↓ IgD expression on splenic B cells
			↓ B-cell proliferation following CD40L stimulation (4/4)	Nearly absent B cells in Peyer’s patches (only in homozygous mice)
			Hypogammaglobulinemia (4/4)	
**T cells**	CD4+ T cells skewed to memory (1/1 evaluated)	↓ levels of CD3e and TCRβ	CD4+ T cells skewed to naive (3/4)	Modest ↑ of T cells in PB
	T cells showed activated phenotypes (1/1 evaluated)	CD4+ T cells skewed to memory (only in homozygous mice)	Imbalanced Th subsets (↓ Th1 (4/4) and Tfh (3/4))	↑ CD4+ T cells in homozygous mice
	Imbalanced Th subsets (↓ Th17 and ↑ Th1*) (1/1 evaluated)	↓ CD8+ T cells	↓ CD40 expression following PMA/Ionomycin stimulation (4/4)	↓ naive T cells (especially CD8+ T cells) in homozygous mice
	↓ levels of CD3 and TCRα/β (1/1 evaluated)			↓ Tfh in Peyer’s patches
***In vitro* characterization**	LOF		LOF	
	DN against AIOLOS^WT^ and IKAROS		DN against AIOLOS^WT^	
**Key molecular mechanism**	Interference against IKAROS *via* formation of heterodimer		Defective CD40/CD40L signal	

↓, decreased; ↑, increased; →, normal.

All patients manifested B-cell lymphopenia (0.63%–3% of total lymphocyte). B cell lineage-committed progenitors and immature B cells were almost absent in the index patient, suggesting a defect in B cell differentiation. However, hypogammaglobulinemia (low IgG and IgM, normal IgA) was only found in one patient (index patient, IgG 605 mg/dL, IgA 272 mg/dL, IgM 20 mg/dL). In other patients, IgG and IgA levels were relatively high (IgG 1328–1714 mg/dL, IgA 251–719 mg/dL) and IgM levels were normal to low (IgM 25–123 mg/dL). IgE level was assessed in one patient and found to be high (1940 IU/L).

T cell abnormality in the patients was characterized by CD4^+^ T cells skewing to memory and activated phenotypes, as 98% and 30% of CD4^+^ T cells were CD45RO^+^ and CD38^+^HLA-DR^+^, respectively. In addition, there was an abnormal Th subset balance, i.e., decreased Th17 and increased Th1* (CD4^+^CD45RO^+^CD161^+^CCR6^+^CXCR3^+^) levels. CD8^+^ T cells in the patients also demonstrated an activated phenotype. In addition, reduced expression levels of CD3 and T-cell receptor (TCR) α/β on T cells were observed.

### AIOLOS N160S Variant

Kuehn et al. reported four patients from a single family with susceptibility to PjP and hypogammaglobulinemia as summarized in **Figure 2** and **Table 1** (27). The index patient contracted PjP at infancy, and hypogammaglobulinemia was discovered upon examination. Her father, paternal aunt, and paternal grandfather were also hypogammaglobulinemic, and two of them had histories of PjP. The medical history of her aunt was remarkable, showing oligoarticular arthritis, and development of CLL and metastatic melanoma in her 30s. All the patients underwent immunoglobulin replacement therapies.

Patients with the AIOLOS^N160S^ variant are characterized by susceptibilities to opportunistic infections. Three out of four patients contracted PjP, whereas recurrent sinopulmonary infections were observed in all the patients. In addition, meningitis in the aunt and sepsis, extensive cutaneous warts, and *Mycobacterium avium* complex lung disease in the grandfather were also observed. No EBV infection was identified within this group of patients. Autoimmunity was not observed in these patients with the AIOLOS^N160S^ variant.

In contrast to the patients with the AIOLOS^G159R^ variant, the patients in this group had normal to high B-cell levels in their peripheral blood (13%–28% of total lymphocytes/234–990 cells/μL) except for the index patient’s grandfather (3% of total lymphocytes/38 cells/μL). Immunoglobulin levels were low in all patients (IgA < 5–10 mg/dL; IgM < 5 mg/dL; IgG 914–1420 mg/dL with immunoglobulin supplementation). Phenotypes of B cells in the patients with AIOLOS^N160S^ were characterized by increased levels of CD21^lo^ B cells, nearly absent memory B cells, and minimal CD23 expressions. Of note, IgM expressions on B cells were decreased in two of the four patients, and the kappa–lambda light chain ratio was skewed toward the kappa chain in three of the four patients.

T cell numbers were normal in the patients with AIOLOS^N160S^. Additionally, naive T cells and recent thymic emigrant levels increased, whereas memory T cells and follicular helper T cell levels decreased in the adult patients. Among all the patients, reduction in Th1 levels was common, whereas the levels of Th2 and Th17 did not reduce.

## Murine Models of AIOLOS Variants

IKZF family of transcription factors is evolutionally well-conserved among species (28), with human and murine Aiolos sharing 87% of the amino acid sequences. In the studies of AIOLOS variants in IEI, knock-in mouse models were produced using CRISPR/Cas9 gene-editing technology to further investigate detailed immunophenotypes and elucidate the pathogenic mechanisms caused by these AIOLOS variants. AIOLOS^G159R^ and AIOLOS^N160S^ in humans correspond to Aiolos^G158R^ and Aiolos^N159S^ in mice, respectively.

### Aiolos-Deficient Mice

Aiolos-deficient mice demonstrated increased pre-B cell fraction and a decrease in mature recirculating B cells in the bone marrow, suggesting the partial differentiation defect of B cells (2). Most splenic B cells in Aiolos-deficient mice were IgD^hi^IgM^lo^ follicular B cells, and marginal zone B cells were absent (29). CD21 expression was downregulated in the B cells. These B cells exhibited activated phenotype, and B-cell receptor (BCR)-stimulated proliferation was also strengthened. Increased germinal center formation and elevated serum IgG and IgE levels were observed in the absence of immunization. Autoantibodies were frequently produced.

In contrast to B cells, T cell development in Aiolos-deficient mice was relatively unaffected. Thymocyte and peripheral T cell populations were comparable to wild-type mice (2). However, augmented TCR-mediated proliferation of T cells was observed. Naive CD4^+^ T cells of Aiolos-deficient mice showed impairment in their differentiation into Th17 cells, producing more IL-2 which is directly regulated by Aiolos. Conversely, Aiolos-deficient CD4^+^ T cells produced more interferon-γ when activated under Th1-polarizing conditions (3).

NK cells were present in the bone marrow and spleen of Aiolos-deficient mice. CD11b^lo^CD27^+^ and CD11b^hi^CD27^+^ NK cell subsets were increased, whereas terminally matured CD11b^hi^CD27^–^ subset was decreased in Aiolos-deficient mice. Aiolos-deficient NK cells showed hyperresponsiveness to activation and proliferation in response to IL-15 stimulation (4).

Spontaneous development of B-cell lymphoma was observed in aging Aiolos-deficient mice (2). Six out of twenty mice developed lymphoproliferations in the peripheral lymphoid organs. Four out of five examined mice had developed B-cell lymphoma, whereas one developed T-cell lymphoproliferation.

### Aiolos G158R Mice

*Aiolos^G158R^* mutant mice showed defects in B-cell developmental with a differentiation blockade at the pre-pro-B cells to pro-B cell and pre-B-cell stages in heterozygous and homozygous mutant mice (26). Splenic B-cell levels were significantly reduced, and all the splenic B-cell subsets, i.e., follicular B cells (B220^+^AA4.1^−^CD23^hi^CD21^int^), marginal zone B cells (B220^+^AA4.1^−^CD23^lo^CD21^+^), and germinal center B cells (B220^+^GL7^+^CD95^+^CD38^–^), were absent in *Aiolos^G158R/G158R^* mice. Splenic B cells decreased in *Aiolos^+/G158R^* as observed in homozygous mutant mice but exhibited a milder phenotype, and B-cell developmental defects in the heterozygous mutant mice was exacerbated with aging.

T-cell phenotypes of *Aiolos^G158R^* mice were also similar to the T cells abnormalities in the patients. CD3ε and TCRβ expression levels on postselection thymocytes and in the later stages of T cells in the thymus and lymph nodes decreased in heterozygous and homozygous *Aiolos^G158R^* mice. Furthermore, CD8^+^ T cells were decreased in the lymph nodes of *Aiolos^G158R^* mice. Additionally, in *Aiolos^G158R/G158R^* mice, CD4^+^ T cells were skewed to memory phenotype, and an increase of CD4^–^CD8^–^ T cells were detected.

### Aiolos N159S Mice

*Aiolos^N159S^* mutant mice showed different B-cell developmental defects than those in *Aiolos^G158R^*, reflecting the different phenotypes of the patients. In contrast to *Aiolos^G158R^* mice, the number of B cells in secondary lymphoid organs and peripheral blood did not decrease in *Aiolos^+/N159S^* mice (27). However, B-cell levels within total lymphocytes decreased in *Aiolos^N159S/N159S^* mice. Characteristic aberrant expression patterns of CD21 and CD23 on mature B cells were recapitulated in the heterozygous and homozygous *Aiolos^N159S^* mice. CD23^hi^CD21^int^ follicular B cells also decreased in the spleen and peripheral blood of *Aiolos^+/N159S^* and *Aiolos^N159S/N159S^* mice. The cell surface expressions of IgM and IgD were altered on the B cells of heterozygous and homozygous *Aiolos^N159S^* mutant mice, but the aberrant pattern differed from the human patients. IgD expression was decreased in the B cells of *Aiolos^+/N159S^* mice, whereas *Aiolos^N159S/N159S^* B cells showed completely abrogated IgD expression. In addition, the B cells in the Peyer’s patches were almost absent in *Aiolos^N159S/N159S^* mice, although residual germinal center B cells were observed. A progressive decrease in IgM and IgA was observed in *Aiolos^+/N159S^* mice. Immunoglobulin of all classes (IgG, IgA, and IgM) decreased in *Aiolos^N159S/N159S^* mice.

T-cell phenotypes of *Aiolos^N159S^* mice showed increased T-cell frequency in the peripheral blood in a gene-dosage dependent manner. CD4^+^ T cells were relatively increased, but the naive CD8^+^ T cells decreased in *Aiolos^N159S/N159S^* mice. In addition, follicular helper T cells were reduced in Peyer’s patches of *Aiolos^+/N159S^* mice and almost absent in the *Aiolos^N159S/N159S^* mice; this may be related to the defects of antibody production in *Aiolos^N159S^* mice.

### Aiolos L161R Mice

Another *Aiolos* mutant mouse (30), a somatic hotspot mutation in AIOLOS (L162R) found in CLL patients, has been recently reported as a human disease model (30) (**Figure 1B**) (25). The B cell-conditional knock-in mouse model harboring the homologous variant L161R (*Cd19-Cre; Aiolos^+/fl^* and *Cd19-Cre; Aiolos^fl/fl^*, hereafter designated as *Aiolos^+/Cd19-L161R^* and *Aiolos^Cd19-L161R/Cd19-L161R^*, respectively) was generated and studied. In terms of binding to canonical consensus sequence and pericentromeric heterochromatin (PC-HC) targeting, the variant was not identified as loss-of-function (27, 30).

B-cell progenitors in the bone marrow were not affected and total splenic B-cell numbers were comparable to wild-type controls. However, there was a decrease in the marginal zone B cells and increase in follicular B cells in *Aiolos^+/Cd19-L161R^* and *Aiolos^Cd19-L161R/Cd19-L161R^* mice. After immunization, an increased fraction of germinal center B cells (B220^+^CD95^+^CD38^–^) was observed in *Aiolos^Cd19-L161R/Cd19-L161R^* mice. Hyperactivation of BCR signaling and overexpression of nuclear factor-κ B (NF-κB) were observed in *Aiolos^Cd19-L161R^* B cells in a gene-dosage dependent manner.

In elderly *Aiolos^Cd19-L161R^* mice (18–24 months of age), 10% of *Aiolos^Cd19-L161R/Cd19-L161R^* and 36% of *Aiolos^+/Cd19-L161R^* mice developed CLL-like disease and occasional splenomegaly.

## Molecular Mechanisms Underlying Immunodeficiency Cause by AIOLOS Variants

### AIOLOS G159R/Aiolos G158R

The AIOLOS^G159R^ variant is characterized *in vitro* as loss of binding to canonical consensus sequence of AIOLOS as shown by electrophoretic mobility shift assay (EMSA) and defective PC-HC foci formation. Furthermore, genome-wide binding of the AIOLOS^G159R^ variant was assessed using AIOLOS^G159R^ ChIP-seq in the AIOLOS-knockout human pre-B cell line NALM-6. In addition to the loss of binding to its physiological binding sites containing the consensus sequences, novel binding sites were detected for the AIOLOS^G159R^ variant enriched with aberrant motifs.

In a study involving AIOLOS^G159R^, a murine model was used to investigate the underlying molecular pathogenesis of immunodeficiency caused by the AIOLOS variant. Given that *Aiolos^G158R^* mice exhibited differentiation defects in B cells at the pre-B cell stage and Aiolos is a transcription factor, the pre-B cells of *Aiolos^+/G158R^* mice were subjected to transcriptome analysis. Genes related to B-cell differentiation were downregulated in the pre-B cells of *Aiolos^+/G158R^* mice. Interestingly, dysregulated genes in the *Aiolos^+/G158R^* pre-B cells had higher frequency of Ikaros bindings in their promoter regions than expected frequency calculated from genome-wide Ikaros bindings.

These findings led to the hypothesis that AIOLOS^G159R^ interferes with IKAROS through the formation of heterodimers, resulting in the dysregulation of IKAROS-regulated genes that are essential for B-cell development. Genome-wide bindings of Aiolos^G158R^ and Ikaros were examined by ChIP-seq experiments in the thymus of *Aiolos^G158R/G158R^* mice. Aiolos^G158R^ was unable to bind to the canonical Aiolos binding motifs; it preferentially bound to aberrant motifs. Genome-wide binding of Ikaros had changed in *Aiolos^G158R/G158R^* thymus as hypothesized. Ikaros was often found to co-bind with *Aiolos^G158R^*, and the binding motifs of Ikaros changed as *Aiolos^G158R^*. *In vitro* PC-HC formation assay showed that the co-transfection of AIOLOS^G159R^ inhibited PC-HC targeting by IKAROS. Additionally, the transcriptional activities of IKAROS and wild-type AIOLOS were inhibited by the co-transfection of AIOLOS^G159R^ in a dominant-negative manner.

Therefore, this evidence suggests that the immune defect caused by AIOLOS^G159R^/Aiolos^G158R^ variants results from the inhibition of IKAROS through the formation of a heterodimer with mutant AIOLOS.

### AIOLOS N160S/Aiolos N159S

*In vitro* characterization of the AIOLOS^N160S^ variant suggested a loss-of-function mutant since EMSA indicated that AIOLOS^N160S^ abolished binding to its canonical consensus sequence. When AIOLOS^N160S^ was co-transfected with wild-type AIOLOS for EMSA, the binding of wild-type AIOLOS was reduced to 20% of the wild-type AIOLOS suggesting the dominant-negative effect of AIOLOS^N160S^ against wild-type AIOLOS. PC-HC formation assay demonstrated that the AIOLOS^N160S^ variant is loss-of-function mutant, and it exerted a dominant-negative effect against wild-type AIOLOS. However, AIOLOS^N160S^ had no dominant-negative effect against IKAROS in PC-HC formation assay.

Considering the clinical similarities between the patients with AIOLOS^N160S^ and those with CD40 ligand (CD40L) deficiency, CD40/CD40L signaling was evaluated in the patients’ B and T cells. CD40 expressions on B cells were not decreased in the patients; however, decreased B cell proliferation upon CD40L plus either anti-IgM, IL-4, or IL-21, was identified in all patients. Moreover, B cells did not differentiate into plasmablasts by the stimulation of CD40L and IL-21. In addition, following PMA and ionomycin stimulation, CD40L expression on activated T cells decreased compared with that in healthy control. These findings suggest that defective CD40L upregulation contributed to the CD40L deficiency-like phenotype, such as susceptibility to *P. jirovecii* infection. The characteristic CD21^lo^ B cells in the patients are likely associated with impaired B-cell activation upon CD40L/CD40 stimulation.

RNA-seq analysis of the naive B cells and T-cell blasts showed several dysregulated genes in the naive B cells of patients with AIOLOS^N160S^. The expressions of several genes related to B-cell development and survival were decreased, including *TNFRSF17* (BCMA), *TNFSF13* (APRIL), and *TNFSF13B* (BAFF). Transcriptomic changes in the T-cell blasts of the patients were associated with immune cell signaling. Additionally, genes involved in the inflammation of respiratory system components, cellular infiltration, and antibody production were downregulated in the patients’ T-cell blasts. Conversely, genes involved in cell survival, homing of cells, chemotaxis, and leukopoiesis were upregulated.

T cells from two patients with AIOLOS^N160S^ were subjected to AIOLOS ChIP-seq to further characterize the effects of N160S mutation. Clustering analysis revealed that the genome-wide binding of AIOLOS in the patients clustered away from that of healthy controls. However, there was no change in the AIOLOS binding motif among the patients and healthy controls. Furthermore, no direct correlation of change in AIOLOS binding in the T cells and differences in the transcriptome of the T-cell blasts were observed among the patients and healthy controls.

## Discussion and Future Direction

The heterozygous AIOLOS^G159R^/Aiolos^G158R^ and AIOLOS^N160S^/Aiolos^N159S^ variants caused adaptive immune defects in humans and mice (**Figure 2**). Both of G159R and N160S variants reside in the ZF2 of the AIOLOS protein, one of four N-terminal ZF that mainly mediates DNA binding. The roles of each ZF are extensively studied in IKAROS. ZF2 and ZF3 of IKAROS are essential in binding to the core IKZF motif, whereas ZF1 and ZF4 determine the specificity to the flanking sequences (31, 32). Within ZF2 and ZF3 of IKAROS, substitutions of the amino acids at -1, 2, 3, and 6 positions in reference to the beginning of the α-helix result in abrogated DNA binding (31, 33, 34). Regarding the positions of substituted amino acids in the AIOLOS variants, G159 and N160 correspond to 2 and 3 position of helical positions, respectively. Thus, missense variants of these amino acids would alter the DNA binding ability of AIOLOS. *In vitro* and *in silico* analyses conducted in the studies of AIOLOS^G159R^ and AIOLOS^N160S^ supported this hypothesis. The fundamental question lies in understanding the clinical and immunological phenotypes of the patients and mouse models; the mutated amino acid is next to each other, but why were there so many differences? For example, the AIOLOS^G159R^ variant is associated with a profound decrease in B cells and possible EBV susceptibility, but two of the three patients were not hypogammaglobulinemic. AIOLOS^N160S^ variant is associated with *P. jirovecii* infection and severe hypogammaglobulinemia, but B cells were not decreased in the patients’ peripheral blood. The phenotypes of the patients were well recapitulated in the mouse models with the homologous variants. Several hypotheses can explain the difference between AIOLOS^G159R^/Aiolos^G158R^ and AIOLOS^N160S^/Aiolos^N159S^, such as 1) the severities of functional defect are different among variants; 2) neomorphic attributes of the variants affect the phenotype; or 3) protein interactions differ among the variants. Both variants were loss-of-function in terms of binding to the canonical consensus sequence and formation of PC-HC and acted in negative-dominance against wild-type AIOLOS. AIOLOS^G159R^ exerted the dominant-negative effect against IKAROS as demonstrated by PC-HC formation and luciferase reporter gene assays but not AIOLOS^N160S^. Thus, the degree of functional damage may be greater in the G159R variant. However, patients with AIOLOS^N160S^ did not necessarily exhibit milder phenotypes. The neomorphic aspects of the mutations may also explain the phenotypic differences. ChIP-seq analyses of AIOLOS^G159R^ and Aiolos^G158R^ detected the novel binding motifs preferentially bound by these variants. In contrast, AIOLOS ChIP-seq in the T cells of patients with AIOLOS^N160S^ did not alter the binding motifs but might have been affected by the bindings of wild-type AIOLOS as half of the AIOLOS protein in the cells would have been the wild-type. In this regard, it is important to compare genome-wide bindings of Aiolos and Ikaros between *Aiolos^G158R/G158R^* and *Aiolos^N159S/N159S^* mice to evaluate how differential bindings by the different AIOLOS variants affect the phenotypes of the patients and mouse models. The current evidence suggests that the newly acquired bindings by AIOLOS^G159R^ and Aiolos^G158R^ do not have cis-regulatory effects on surrounding genes but rather work as sequestration of IKAROS to non-physiological binding sites. Although the N-terminal ZFs (ZF1-4) of AIOLOS is known to mediate DNA binding, there is a possibility that the variants in these ZFs also affect protein–protein interaction. The alteration of protein–protein interactions by each variant will be the subject of the future research.

Alternative mRNA splicing of IKZF molecules results in multiple isoforms with differential patterns of domains present in each isoform. The isoforms lacking the whole DNA binding domain or core binding ZFs (ZF2 and ZF3 for Ikaros) are designated as dominant-negative isoforms. Dominant-negative isoforms lack DNA binding ability while maintaining dimerizing capacity, and they inhibit the DNA binding of dimerizing partners. The dominant-negative isoforms of IKZF proteins can be detected in normal lymphocytes (35–37). However, the specific roles of these dominant-negative isoforms in the lymphocyte development are still not known. The expressions of dominant-negative isoforms of IKZF molecules are also observed in various hematologic malignancies (37–41). *IKAROS^N159S^* somatic mutation is also found in ALL (42). Contribution of dominant-negative IKAROS to the leukemogenesis is further supported by the observations of spontaneous development of T-cell leukemia and lymphoma in the heterozygous *Ikaros* dominant-negative (ΔZF1-3) mice and the mice harboring heterozygous *Ikaros^H191R^* mutation (43, 44). Conditional deletion of *Ikaros* exon 5 (coding ZF2 and ZF3) in common lymphoid progenitors (*Cd2-Cre; Ikaros E5^fl/fl^*) also developed T-lymphoid malignancies (45). Pre-B cells of Ikaros *Cd2-Cre; Ikaros E5^fl/fl^* mice underwent leukemic transformation when transplanted to immunodeficient NOD SCID gamma (NSG) mouse. Ikaros-deficient mice lack B cells, NK cell, and fetal T cell, while T cells develop postnatally (46). On the other hand, *Ikaros* dominant-negative (ΔZF1-3) mice and mice with ENU-mediated missense mutation in ZF3 (H191R) demonstrate severer lymphocyte developmental defect characterized by absent T, B, and NK cells (44, 47). *Ikaros^H191R/H191R^* fetal liver cells demonstrated defect in terminal myelopoiesis and erythropoiesis. Moreover, *Ikaros^H191R/H191R^* mice were fatally lethal (44). The severer phenotypes observed in *Ikaros* dominant-negative mice when compared to Ikaros-deficient mice have been hypothesized as the results of dominant-negative effect against other IKZF molecules by the mutant Ikaros. The studies of AIOLOS variants provided counterpart of this hypothesis. Heterozygous and homozygous *Aiolos^G158R^* and *Aiolos^N159S^* variants resulted in severer lymphocyte developmental defects when compared to Aiolos-deficient mice. These observations indicate dominant-negative effect of Aiolos^G158R^ and Aiolos^N159S^ variants against other IKZF molecules. Precisely, it is not clear whether the phenotypes of these mutant mice solely resulted from the dominant-negative effects against other molecules or partially due to neomorphic function by the variants. Also, the roles of each ZF of Aiolos in lymphocyte development have not been studied. In this regard, it is essential to compare the phenotypes of *Aiolos* dominant-negative (ΔZF1-4) mice, mice lacking individual Aiolos ZFs, and the missense *Aiolos^G158R^* and *Aiolos^N159S^* mutant mice.

One interesting feature of the AIOLOS^N160S^ variant is its susceptibility to PjP. The homologous variant in IKAROS (N159S) is associated with similar clinical and immunological phenotypes, including PjP susceptibility (14, 15). B cell levels in patients with AIOLOS^N160S^ were normal to high, whereas class-switched memory B cell and plasmablast levels were low. In addition, CD23 and CD21 expressions were decreased on the B cells. In contrast, B cells in IKAROS^N159S^ patients were almost absent. Both variants were associated with severe hypogammaglobulinemia. T cell abnormalities were characterized by increased naive CD4^+^ T cells and impaired Th polarization (decreased Th1 in AIOLOS^N160S^ and decreased Th1, Th2, and Th17 in IKAROS^N159S^ patients) in both AIOLOS^N160S^ and IKAROS^N159S^ patients. Impaired CD40/CD40L signals were the cause of PjP susceptibility in patients with AIOLOS^N160S^ but no CD40L signal defects were reported in patients with IKAROS^N159S^. The precise mechanism underlying PjP susceptibility in patients with AIOLOS^N160S^ and IKAROS^N159S^ should be investigated in future studies.

The development of hematologic malignancies is a common clinical characteristic of IEI caused by AIOLOS variants. EBV-associated lymphoma was observed in two of the three patients with AIOLOS^G159R^, and CLL was observed in one of the four patients with AIOLOS^N160S^. The tendency to develop hematologic malignancy in patients with the AIOLOS variants is plausible as they were observed in patients with IKAROS and HELIOS deficiencies (17, 19). Additionally, germline variants of IKAROS were also detected in cohorts of patients with B-ALL (48). Although, the precise mechanism underlying tumorigenesis in patients with the AIOLOS variants remains unknown. Two patients with AIOLOS^G159R^ developed EBV-associated lymphoma, whereas another patient presented with chronic and recurrent EBV infections. Thus, the underlying cause of lymphomagenesis is possibly EBV susceptibility. On contrast, somatic loss-of-function AIOLOS mutations were reported in B-ALL (22). Moreover, aged Aiolos-null mice develop B-cell lymphoma (2). These studies and reports of IEI caused by AIOLOS variants indicate a tumor-suppressive role of AIOLOS and the development of hematologic malignancies following the functional deterioration of AIOLOS. Alternatively, altered interaction with other IKZF molecules and heteromerizing partners or neomorphic features of the AIOLOS variants may have led to cancer development in the patients. The somatic *AIOLOS^L162R^* mutation was repeatedly reported in CLL (25). AIOLOS^L162R^ variant was not proven to be loss-of-function in DNA binding and PC-HC targeting abilities (27, 30). Studies on the murine homologous variant *Aiolos^L161R^* suggested that this variant enhanced BCR and NF-κB signaling, contributing to the CLL-like disease development in aged mice. It is not clear if CLL development in patients with AIOLOS^N160S^ shared the same molecular mechanism as in AIOLOS^L162R^/Aiolos^L161R^.

Studies on AIOLOS^G159R^/Aiolos^G158R^ demonstrated that these variants can cause pathogenicity through heterodimerization with Ikaros, interfering with the physiological function of Ikaros. Seeking therapeutic potential for the patients with IEI caused by AIOLOS variants, we performed gene therapy for *Aiolos^G158R^* mice. A small indel was introduced before the dimerizing ZF (ZF5) of the mutant Aiolos allele, designated as *Aiolos^G158R:Δc-ZF^*, using CRISPR/Cas9 gene-editing technology (26). This mutant theoretically had a DNA-binding domain, but it could not form a dimer with wild-type Aiolos or Ikaros and other dimerizing partners. The *Aiolos^+/G158R:Δc-ZF^* mice recovered the B-cell levels in the peripheral lymphoid tissues and early B-cell development in the bone marrow was normalized. T cell abnormalities that were observed in *Aiolos^+/G158R^* mice were also normalized. These findings suggest the therapeutic potential of gene therapy and small molecules that interfere with the AIOLOS–IKAROS interaction. However, applying this strategy to the patients will be challenging. Germinal center B cells increased in mice with biallelic C-terminal deletion in the Aiolos, as previously reported in Aiolos-deficient mice. Considering autoimmunity and spontaneous lymphoma development in *Aiolos*-knockout mice, selective inhibition of mutant AIOLOS allele/protein would be ideal strategy.

Increasing number of novel IEIs is being reported with the advancement of genetic diagnostic technologies. However, most newly described IEIs are sporadic and are often confined to a handful of patients. For example, the first reports of IEI caused by AIOLOS variants include only seven patients in two families. Although these studies address extremely rare disease, detailed phenotyping of the patients and thorough understanding of underlying mechanism using animal models can provide insights into the unknown but essential aspects of Aiolos in the development and function of lymphocytes. Future studies should address the differences in the phenotypes of immunodeficiency caused by AIOLOS variants and novel therapeutic approaches that specifically inhibit mutant AIOLOS proteins without interfering with the unaffected allele and other heterodimerizing partners should be developed.

## Author Contributions

MY and TM wrote the manuscript. All authors contributed to the article and approved the submitted version.

## Funding

This work was supported by JSPS Grant-in-Aid for Young Scientists JP20K16884 (MY) and Scholarship grant 91AA191397 (TM).

## Conflict of Interest

The authors declare that the research was conducted in the absence of any commercial or financial relationships that could be construed as a potential conflict of interest.

The handling editor declared a past collaboration with the authors MY and TM.

## Publisher’s Note

All claims expressed in this article are solely those of the authors and do not necessarily represent those of their affiliated organizations, or those of the publisher, the editors and the reviewers. Any product that may be evaluated in this article, or claim that may be made by its manufacturer, is not guaranteed or endorsed by the publisher.

## References

[1] Morgan B Sun L Avitahl N Andrikopoulos K Ikeda T Gonzales E. Aiolos, a Lymphoid Restricted Transcription Factor That Interacts With Ikaros to Regulate Lymphocyte Differentiation. EMBO J (1997) 16:2004–13. doi: 10.1093/emboj/16.8.2004 PMC1169803 9155026

[2] Wang J-H Avitahl N Cariappa A Friedrich C Ikeda T Renold A. Aiolos Regulates B Cell Activation and Maturation to Effector State. Immunity (1998) 9:543–53. doi: 10.1016/s1074-7613(00)80637-8 9806640

[3] Quintana FJ Jin H Burns EJ Nadeau M Yeste A Kumar D. Aiolos Promotes TH17 Differentiation by Directly Silencing Il2 Expression. Nat Immunol (2012) 13:770–7. doi: 10.1038/ni.2363 PMC3541018 22751139

[4] Holmes ML Huntington ND Thong RP Brady J Hayakawa Y Andoniou CE. Peripheral Natural Killer Cell Maturation Depends on the Transcription Factor Aiolos. EMBO J (2014) 33:2721–34. doi: 10.15252/embj.201487900 PMC4282578 25319415

[5] Thompson EC Cobb BS Sabbattini P Meixlsperger S Parelho V Liberg D. Ikaros DNA-Binding Proteins as Integral Components of B Cell Developmental-Stage-Specific Regulatory Circuits. Immunity (2007) 26:335–44. doi: 10.1016/j.immuni.2007.02.010 17363301

[6] Ma S Pathak S Mandal M Trinh L Clark MR Lu R. Ikaros and Aiolos Inhibit Pre-B-Cell Proliferation by Directly Suppressing C-Myc Expression. Mol Cell Biol (2010) 30:4149–58. doi: 10.1128/mcb.00224-10 PMC2937562 20566697

[7] Harker N Naito T Cortes M Hostert A Hirschberg S Tolaini M. The Cd8α Gene Locus Is Regulated by the Ikaros Family of Proteins. Mol Cell (2002) 10:1403–15. doi: 10.1016/s1097-2765(02)00711-6 12504015

[8] Read KA Powell MD Baker CE Sreekumar BK Ringel-Scaia VM Bachus H. Integrated STAT3 and Ikaros Zinc Finger Transcription Factor Activities Regulate Bcl-6 Expression in CD4+ Th Cells. J Immunol (2017) 199:2377–87. doi: 10.4049/jimmunol.1700106 PMC5657606 28848064

[9] Yamashita M Morio T. Inborn Errors of IKAROS and AIOLOS. Curr Opin Immunol (2021) 72:239–48. doi: 10.1016/j.coi.2021.06.010 34265590

[10] Georgopoulos K. The Making of a Lymphocyte: The Choice Among Disparate Cell Fates and the IKAROS Enigma. Gene Dev (2017) 31:439–50. doi: 10.1101/gad.297002.117 PMC5393059 28385788

[11] Heizmann B Kastner P Chan S. The Ikaros Family in Lymphocyte Development. Curr Opin Immunol (2018) 51:14–23. doi: 10.1016/j.coi.2017.11.005 29278858

[12] Goldman FD Gurel Z Al-Zubeidi D Fried AJ Icardi M Song C. Congenital Pancytopenia and Absence of B Lymphocytes in a Neonate With a Mutation in the Ikaros Gene. Pediatr Blood Amp Cancer (2011) 58:591–7. doi: 10.1002/pbc.23160 PMC3161153 21548011

[13] Kuehn HS Boisson B Cunningham-Rundles C Reichenbach J Stray-Pedersen A Gelfand EW. Loss of B Cells in Patients With Heterozygous Mutations in IKAROS. N Engl J Med (2016) 374:1032–43. doi: 10.1056/nejmoa1512234 PMC4836293 26981933

[14] Hoshino A Okada S Yoshida K Nishida N Okuno Y Ueno H. Abnormal Hematopoiesis and Autoimmunity in Human Subjects With Germline IKZF1 Mutations. J Allergy Clin Immun (2017) 140:223–31. doi: 10.1016/j.jaci.2016.09.029 27939403

[15] Boutboul D Kuehn HS de Wyngaert ZV Niemela JE Callebaut I Stoddard J. Dominant-Negative IKZF1 Mutations Cause a T, B, and Myeloid Cell Combined Immunodeficiency. J Clin Invest (2018) 128:3071–87. doi: 10.1172/jci98164 PMC6026000 29889099

[16] Yoshida N Sakaguchi H Muramatsu H Okuno Y Song C Dovat S. Germline IKAROS Mutation Associated With Primary Immunodeficiency That Progressed to T-Cell Acute Lymphoblastic Leukemia. Leukemia (2017) 31:1221–3. doi: 10.1038/leu.2017.25 28096536

[17] Kuehn HS Nunes-Santos CJ Rosenzweig SD. IKAROS-Associated Diseases in 2020: Genotypes, Phenotypes, and Outcomes in Primary Immune Deficiency/Inborn Errors of Immunity. J Clin Immunol (2021) 41:1–10. doi: 10.1007/s10875-020-00936-x 33392855

[18] Shahin T Kuehn HS Shoeb MR Gawriyski L Giuliani S Repiscak P. Germline Biallelic Mutation Affecting the Transcription Factor Helios Causes Pleiotropic Defects of Immunity. Sci Immunol (2021) 6:eabe3981. doi: 10.1126/sciimmunol.abe3981 34826259 PMC7612971

[19] Hetemäki I Kaustio M Kinnunen M Heikkilä N Keskitalo S Nowlan K. Loss-Of-Function Mutation in IKZF2 Leads to Immunodeficiency With Dysregulated Germinal Center Reactions and Reduction of MAIT Cells. Sci Immunol (2021) 6:eabe3454. doi: 10.1126/sciimmunol.abe3454 34826260

[20] Shahin T Mayr D Shoeb MR Kuehn HS Hoeger B Giuliani S. Identification of Germline Monoallelic Mutations in IKZF2 in Patients With Immune Dysregulation. Blood Adv (2021). doi: 10.1182/bloodadvances.2021006367 PMC9006292 34920454

[21] Lentaigne C Greene D Sivapalaratnam S Favier R Seyres D Thys C. Germline Mutations in the Transcription Factor IKZF5 Cause Thrombocytopenia. Blood (2019) 134:2070–81. doi: 10.1182/blood.2019000782 31217188

[22] Mullighan CG Goorha S Radtke I Miller CB Coustan-Smith E Dalton JD. Genome-Wide Analysis of Genetic Alterations in Acute Lymphoblastic Leukaemia. Nature (2007) 446:758–64. doi: 10.1038/nature05690 17344859

[23] Duhamel M Arrouss I Merle-Béral H Rebollo A. The Aiolos Transcription Factor Is Up-Regulated in Chronic Lymphocytic Leukemia. Blood (2008) 111:3225–8. doi: 10.1182/blood-2007-09-113191 18184862

[24] Billot K Soeur J Chereau F Arrouss I Merle-Béral H Huang M-E. Deregulation of Aiolos Expression in Chronic Lymphocytic Leukemia Is Associated With Epigenetic Modifications. Blood (2011) 117:1917–27. doi: 10.1182/blood-2010-09-307140 21139082

[25] Landau DA Tausch E Taylor-Weiner AN Stewart C Reiter JG Bahlo J. Mutations Driving CLL and Their Evolution in Progression and Relapse. Nature (2015) 526:525–30. doi: 10.1038/nature15395 PMC4815041 26466571

[26] Yamashita M Kuehn HS Okuyama K Okada S Inoue Y Mitsuiki N. A Variant in Human AIOLOS Impairs Adaptive Immunity by Interfering With IKAROS. Nat Immunol (2021) 22:893–903. doi: 10.1038/s41590-021-00951-z 34155405 PMC8958960

[27] Kuehn HS Chang J Yamashita M Niemela JE Zou C Okuyama K. T and B Cell Abnormalities, Pneumocystis Pneumonia, and Chronic Lymphocytic Leukemia Associated With an AIOLOS Defect in Patients. J Exp Med (2021) 218:e20211118. doi: 10.1084/jem.20211118 34694366 PMC8548914

[28] John LB Ward AC. The Ikaros Gene Family: Transcriptional Regulators of Hematopoiesis and Immunity. Mol Immunol (2011) 48:1272–8. doi: 10.1016/j.molimm.2011.03.006 21477865

[29] Cariappa A Tang M Parng C Nebelitskiy E Carroll M Georgopoulos K. The Follicular Versus Marginal Zone B Lymphocyte Cell Fate Decision Is Regulated by Aiolos, Btk, and CD21. Immunity (2001) 14:603–15. doi: 10.1016/s1074-7613(01)00135-2 11371362

[30] Lazarian G Yin S ten Hacken E Sewastianik T Uduman M Font-Tello A. A Hotspot Mutation in Transcription Factor IKZF3 Drives B Cell Neoplasia *via* Transcriptional Dysregulation. Cancer Cell (2021) 39:380–393.e8. doi: 10.1016/j.ccell.2021.02.003 33689703 PMC8034546

[31] Cobb BS Morales-Alcelay S Kleiger G Brown KE Fisher AG Smale ST. Targeting of Ikaros to Pericentromeric Heterochromatin by Direct DNA Binding. Gene Dev (2000) 14:2146–60. doi: 10.1101/gad.816400 PMC316893 10970879

[32] Schjerven H McLaughlin J Arenzana TL Frietze S Cheng D Wadsworth S. Differential Regulation of Lymphopoiesis and Leukemogenesis by Individual Zinc Fingers of Ikaros. Nat Immunol (2013) 14:1073–83. doi: 10.1038/ni.2707 PMC3800053 24013668

[33] Payne MA. Zinc Finger Structure-Function in Ikaros. World J Biol Chem (2011) 2:161–6. doi: 10.4331/wjbc.v2.i6.161 PMC3135863 21765982

[34] Klug A. The Discovery of Zinc Fingers and Their Applications in Gene Regulation and Genome Manipulation. Annu Rev Biochem (2010) 79:213–31. doi: 10.1146/annurev-biochem-010909-095056 20192761

[35] Molnár A Georgopoulos K. The Ikaros Gene Encodes a Family of Functionally Diverse Zinc Finger DNA-Binding Proteins. Mol Cell Biol (1994) 14:8292–303. doi: 10.1128/mcb.14.12.8292 PMC359368 7969165

[36] Liippo J Nera K Veistinen E Lähdesmäki A Postila V Kimby E. Both Normal and Leukemic B Lymphocytes Express Multiple Isoforms of the Human Aiolos Gene. Eur J Immunol (2001) 31:3469–74. doi: 10.1002/1521-4141(200112)31:12<3469::aid-immu3469>3.0.co;2-g 11745366

[37] Caballero R Setien F Lopez-Serra L Boix-Chornet M Fraga MF Ropero S. Combinatorial Effects of Splice Variants Modulate Function of Aiolos. J Cell Sci (2007) 120:2619–30. doi: 10.1242/jcs.007344 17646674

[38] Sun L Heerema N Crotty L Wu X Navara C Vassilev A. Expression of Dominant-Negative and Mutant Isoforms of the Antileukemic Transcription Factor Ikaros in Infant Acute Lymphoblastic Leukemia. Proc Natl Acad Sci (1999) 96:680–5. doi: 10.1073/pnas.96.2.680 PMC15196 9892693

[39] Nishii K Katayama N Miwa H Shikami M Usui E Masuya M. Non-DNA-Binding Ikaros Isoform Gene Expressed in Adult B-Precursor Acute Lymphoblastic Leukemia. Leukemia (2002) 16:1285–92. doi: 10.1038/sj.leu.2402533 12094252

[40] Tokunaga K Yamaguchi S Iwanaga E Nanri T Shimomura T Suzushima H. High Frequency of IKZF1 Genetic Alterations in Adult Patients With B-Cell Acute Lymphoblastic Leukemia. Eur J Haematol (2013) 91:201–8. doi: 10.1111/ejh.12155 23751147

[41] Zhao S Liu W Li Y Liu P Li S Dou D. Alternative Splice Variants Modulates Dominant-Negative Function of Helios in T-Cell Leukemia. PloS One (2016) 11:e0163328. doi: 10.1371/journal.pone.0163328 27681508 PMC5040427

[42] Lana T de Lorenzo P Bresolin S Bronzini I den Boer ML Cavé H. Refinement of IKZF1 Status in Pediatric Philadelphia-Positive Acute Lymphoblastic Leukemia. Leukemia (2015) 29:2107–10. doi: 10.1038/leu.2015.78 25778098

[43] Winandy S Wu P Georgopoulos K. A Dominant Mutation in the Ikaros Gene Leads to Rapid Development of Leukemia and Lymphoma. Cell (1995) 83:289–99. doi: 10.1016/0092-8674(95)90170-1 7585946

[44] Papathanasiou P Perkins AC Cobb BS Ferrini R Sridharan R Hoyne GF. Widespread Failure of Hematolymphoid Differentiation Caused by a Recessive Niche-Filling Allele of the Ikaros Transcription Factor. Immunity (2003) 19:131–44. doi: 10.1016/s1074-7613(03)00168-7 12871645

[45] Joshi I Yoshida T Jena N Qi X Zhang J Etten RAV. Ikaros Mutation Confers Integrin-Dependent Pre-B Cell Survival and Progression to Acute Lymphoblastic Leukemia. Nat Immunol (2014) 15:294–304. doi: 10.1038/ni.2821 24509510 PMC4494688

[46] Wang J-H Nichogiannopoulou A Wu L Sun L Sharpe AH Bigby M. Selective Defects in the Development of the Fetal and Adult Lymphoid System in Mice With an Ikaros Null Mutation. Immunity (1996) 5:537–49. doi: 10.1016/s1074-7613(00)80269-1 8986714

[47] Georgopoulos K Bigby M Wang J-H Molnar A Wu P Winandy S. The Ikaros Gene is Required for the Development of All Lymphoid Lineages. Cell (1994) 79:143–56. doi: 10.1016/0092-8674(94)90407-3 7923373

[48] Churchman ML Qian M te Kronnie G Zhang R Yang W Zhang H. Germline Genetic IKZF1 Variation and Predisposition to Childhood Acute Lymphoblastic Leukemia. Cancer Cell (2018) 33:937–48.e8. doi: 10.1016/j.ccell.2018.03.021 29681510 PMC5953820

